# Limited Evidence for Depth Specialism in Isolated Seamount Reef Predators

**DOI:** 10.1002/ece3.72044

**Published:** 2025-08-28

**Authors:** B. J. Cresswell, G. F. Galbraith, A. Barnett, H. B. Harrison, G. P. Jones, E. C. McClure, T. J. R. Quimpo, A. S. Hoey

**Affiliations:** ^1^ College of Science and Engineering James Cook University Townsville Queensland Australia; ^2^ AIMS@JCU Australian Institute of Marine Science Townsville Queensland Australia; ^3^ Biopixel Oceans Foundation Cairns Queensland Australia; ^4^ Marine Data Technology Hub, College of Science and Engineering James Cook University Townsville Queensland Australia; ^5^ School of Biological Sciences University of Bristol Bristol UK

**Keywords:** depth, MCE, pinnacle, predatory fishes, seamount coral reef

## Abstract

Gradients in light, temperature and hydrodynamics associated with water depth are important determinants of ecological communities in marine environments. While depth specialism in coral reef fishes has been extensively studied in shallow (< 30 m) coastal reef systems, less is known about how depth‐associated drivers operate over the larger depth ranges on isolated pinnacle and seamount reef systems, which are known to support abundant assemblages of predatory fishes. Using remotely operated vehicles, we surveyed predatory fish assemblages across a 100 m depth gradient on three seamount reefs in the Coral Sea. We tested for declines in abundance and diversity, as well as differences in assemblage structure of predatory fishes among depth strata. Species richness and abundance decreased significantly with depth, with predator abundance declining fourfold between the shallowest (5 m) and deepest (95 m) depths surveyed, while species richness was halved. Despite this, compositional differences among depth zones were minimal, with most taxa spanning the full depth range, suggesting adaptations to the limited horizontal habitat available on seamounts. Depth‐associated shifts in taxonomic composition were primarily attributed to a single predator family, reef sharks (Carcharhinidae), which increased in abundance at mesophotic depths. The capacity of a large number of predatory fish taxa to utilize a wide range of depths allows these organisms to access favoured thermal environments and may be a potential resilience mechanism under future environmental change. Further studies are needed to assess the implications of depth use for predator behaviour, trophodynamics and conservation strategies.

## Graphical abstract

We investigated how depth influences predatory fish assemblages on seamount reefs in the Coral Sea using remotely operated vehicle surveys across a 100 m depth gradient. Predator abundance and species richness declined significantly with depth; yet assemblage composition remained largely consistent, suggesting many taxa utilise a broad depth range. These findings highlight the adaptability of seamount predators to depth‐associated environmental gradients, with implications for their resilience under future environmental change.

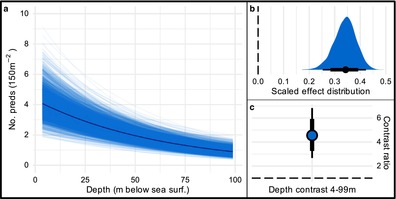

## 1 Introduction

The distribution of ecological communities varies along environmental gradients associated with altitude (Willig and Presley 2016), latitude (Fischer 1960) and depth (Costello and Chaudhary 2017). On coral reefs, one of the most important environmental gradients is depth, along which other important physical parameters like light, temperature and hydrodynamic forces vary predictably (Van den Hoek et al. 1978; Laverick et al. 2020). These gradients in environmental variables are key drivers of the abundance and composition of reef habitat‐forming organisms, such as corals and algae (Done 1982a, 1982b; Williams 1991), and of mobile reef‐associated taxa, such as invertebrates and fishes (MacDonald et al. 2016; Coleman et al. 2018; Mecho et al. 2021). Early studies identified depth as a key driver of the composition of reef fish communities (Leis 1986; Williams 1991; McGehee 1994; Gutiérrez 1998), and a substantial body of work has since demonstrated that fish abundance and species richness generally decline with depth (Brokovich et al. 2008; Jankowski et al. 2015; Pinheiro, MacDonald, Quimbayo, et al. 2023). However, the majority of research on depth patterns in tropical reef fishes has been conducted in the shallows (< 30 m) (Menza et al. 2007; Pyle and Copus 2019; Laverick et al. 2020), with the vast majority being conducted at depths less than 15 m (Roberts and Ormond 1987; McGehee 1994; Meekan et al. 1995; Núñez Lara and Arias González 1998; Khalaf and Kochzius 2002; González‐Sansón et al. 2009). Below these shallow coral reef habitats lie Mesophotic Coral Ecosystems (MCEs); light‐dependent communities that exist between 30 and 150 m (Puglise et al. 2009; Hinderstein et al. 2010).

MCEs are an extension of shallow water coral reefs (Bongaerts 2022), where environmental conditions transition along the depth gradient through shallow (< 30 m), upper‐mesophotic (30–60 m) and lower‐mesophotic zones (60–150 m). Across this gradient, species turnover is a key driver of low species overlap, resulting in unique biological communities at different depths (Rocha et al. 2018). Definitive depth boundaries between these zones can vary globally, but broadly, the upper depths of MCEs represent the start of a transition to mesophotic communities, and the lower limits of MCEs represent the maximum depth that scleractinian corals can survive (Bongaerts 2022). For fishes in MCEs, there is strong evidence for patterns of declines in both species richness and abundance with depth (Thresher and Colin 1986; Andradi‐Brown et al. 2016; Cooper et al. 2019; Scott et al. 2022; Pinheiro, MacDonald, Quimbayo, et al. 2023). However, as with shallow reefs, variation exists within this general pattern, and exceptions to monotonic declines in diversity and abundance can arise because assemblages are usually structured along depth gradients (Lecchini et al. 2003), with different taxa occupying different preferred depth zones (Waldner and Robertson 1980; Srinivasan 2003). Notably, at depths where both deep and shallow water assemblages overlap, peaks in both species richness and abundance can occur (Thresher and Colin 1986; Brokovich et al. 2008; Fukunaga et al. 2016; Pyle et al. 2019; Hoban et al. 2023). Peaks in abundance can also arise from increased numbers of certain guilds, such as planktivores, which frequently aggregate on reef slopes and walls (Hamner et al. 1988; Meekan et al. 1995; Khalaf and Kochzius 2002).

Recent decades have seen increased research attention on MCEs thanks to developments in both technical diving (Pyle 2000), more recently, diver‐independent methods such as remotely operated vehicles (ROVs) (Sward et al. 2019). However, the study of MCEs remains logistically challenging (Puglise and Colin 2016; Bongaerts 2022), particularly for highly mobile taxa like reef fishes, many of which are yet to be scientifically described (Pyle et al. 2019). Furthermore, relative to coastal fringing and barrier reefs, certain coral reef morphologies remain poorly investigated, such as those found on isolated tropical seamounts and pinnacles. Coral reefs on these distinct bathymetric features can exist on both summits in the well‐lit euphotic zone (< 30 m) and also down the surrounding slopes into mesophotic depth ranges (Pérez‐Rosales et al. 2021; Galbraith et al. 2024), providing substantial habitat for coral reef organisms across a wide depth gradient.

The potential for depth‐driven patterns in species abundance and assemblages is thus substantial, with emerging evidence that communities on these structures do not conform to established depth‐diversity patterns (Bridges et al. 2022). In particular, the unique hydrological dynamics of these isolated bathymetric features may be a driver of these differences (Genin 2004; Genin and Dower 2007; Pitcher and Bulman 2007; White et al. 2007; Galbraith et al. 2023; Robinson et al. 2024), where allochthonous subsidies of nutrients and plankton sustain abundant and unique assemblages of fishes compared to those of nearby shallow, coastal reefs (Letessier et al. 2016; Galbraith et al. 2021; Baletaud et al. 2023). Such energy subsidies, in combination with complex and efficient energy pathways, can support top‐heavy trophic webs (‘inverted pyramids’) on seamount and pinnacle reefs, potentially acting as ‘predator magnets’ (Morato et al. 2010; Cresswell et al. 2023). Predators are key organisms in coral reef food webs, where they can drive the structure of whole fish assemblages (Carr et al. 2002; Hixon and Webster 2002; Hixon and Jones 2005 and reviews in Hixon 1991, 2015). In the Indo‐Pacific, recent work has suggested that seamounts and other isolated reefs represent important refuges for marine predators, and globally, these habitats should be considered key conservation priorities for pelagic biodiversity (Letessier et al. 2019; Thompson et al. 2024). Despite the potential importance of these habitats in sustaining predatory fish assemblages, no studies have examined how predator assemblages on seamount or pinnacle reefs vary with depth.

In the present study, we surveyed predatory fish assemblages on three remote seamount reefs in the Coral Sea, SW Pacific, across a 100 m depth range, from the surface to the lower mesophotic. We address the following questions and hypotheses:

1. Does predatory fish abundance diminish with depth on seamount reefs? These structures are known to support abundant assemblages of predators; however, depth patterns in predatory fishes on other reef types suggest declining abundance with depth across both the shallows and mesophotic depths. We therefore predicted that predator abundance would also decline with depth on seamount reefs.
2. Does increasing depth correspond with declining diversity in predatory fish communities on seamount reefs? Existing work suggests declines in species richness with depth across a wide variety of taxa; so we predicted similar patterns for predatory fish assemblages on seamount reefs.
3. Do different depth zones support different assemblage compositions? If so, what families are associated with shallow water and what families are characteristic of the deep reef slopes. As for coastal reef systems, we expected to find some evidence of depth specialism, with most taxa having particular depth preferences.

## 2 Materials and Methods

### 2.1 Study System

**FIGURE 1:**
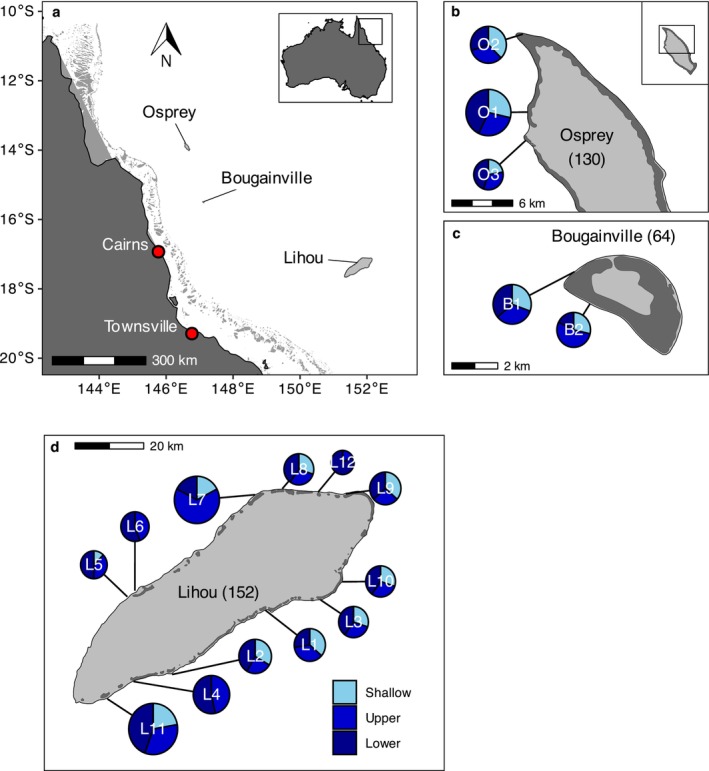
(a) Location of Osprey, Bougainville and Lihou reefs in the central and northern Coral Sea. (b) ROV survey sites at Osprey Reef (3 sites). (c) Bougainville Reef (2 sites), (d). Lihou Reef (12 sites). Total numbers of transects per reef are indicated in parentheses and relative reef transect numbers indicated by pie size. Proportions of transect conducted at depth zones per site are indicated by pie colour, with depth zone coded in blue shades. Shallow = 0–30 m, Upper (mesophotic) = 31–60 m, Lower (mesophotic) = 61–100 m depth.

The study was conducted on three seamount reefs in the central and northern Coral Sea (Figure 1): Lihou (17.1° S, 151.9° E), Osprey (13.9° S, 146.6° E) and Bougainville (15.5° S, 147.1° E) reefs. These reefs represent a range of sizes, with Bougainville being the smallest (1 kha outer reef surface area), Lihou the largest (26 kha outer reef area) and Osprey of intermediate size (6.7 kha outer reef area). The Coral Sea is among the 4% of the world's oceans that remain relatively unaffected by direct anthropogenic disturbances (Halpern et al. 2008). In addition, with the exception of a small portion of the northwest edge of Bougainville Reef, these three locations are further protected by existing almost entirely within no‐take marine reserve boundaries of Australia's Coral Sea Marine Park (CSMP). These oceanic structures are typical of the seamounts of the wider region, extending from depths of around 2000–3000 m to the sea surface and being located in highly oligotrophic, clear ocean waters. These conditions allow light to penetrate to extended depths, and coral reef habitats are known to extend to over 100 m on these reefs (Galbraith et al. 2024).

### 2.2 Data Collection and Processing

Between February and October 2021, three voyages were undertaken to the three study reefs, and remotely operated vehicles (ROVs) deployed to conduct 346 belt video transect surveys of fish communities, from 0 to 100 m depth. The vehicle model used was a BlueROV2 R2 unit (Blue Robotics 2021) in heavy configuration (8 × T200 thrusters). Full ROV survey protocols can be found in Galbraith et al. (2022). Briefly, the ROV was equipped with a calibrated stereo video system (SVS) consisting of either 2 × Paralenz DC + or GoPro8 cameras in aluminium ACTIONPRO T‐housings rated to 300 m and configured with a lateral separation of 400 mm and a convergence angle of 5°. Cameras were configured to high‐definition (minimum 1080p, 60fps), wide‐angle, low‐light optimised settings. Calibration was conducted using the CAL software (SeaGIS Pty Ltd) and all other SVS methods followed Goetze et al. (2019). At each site, the ROV was deployed and descended to the maximum intended depth for that site. The targeted maximum depth was 100 m, corresponding to the approximate extent of the euphotic zone in the Coral Sea (Ceccarelli et al. 2013). Once at maximum target depth, timed video transects were conducted along the reef wall/slope with a crew member making a note of direction, time, depth and other metadata.

**FIGURE 2:**
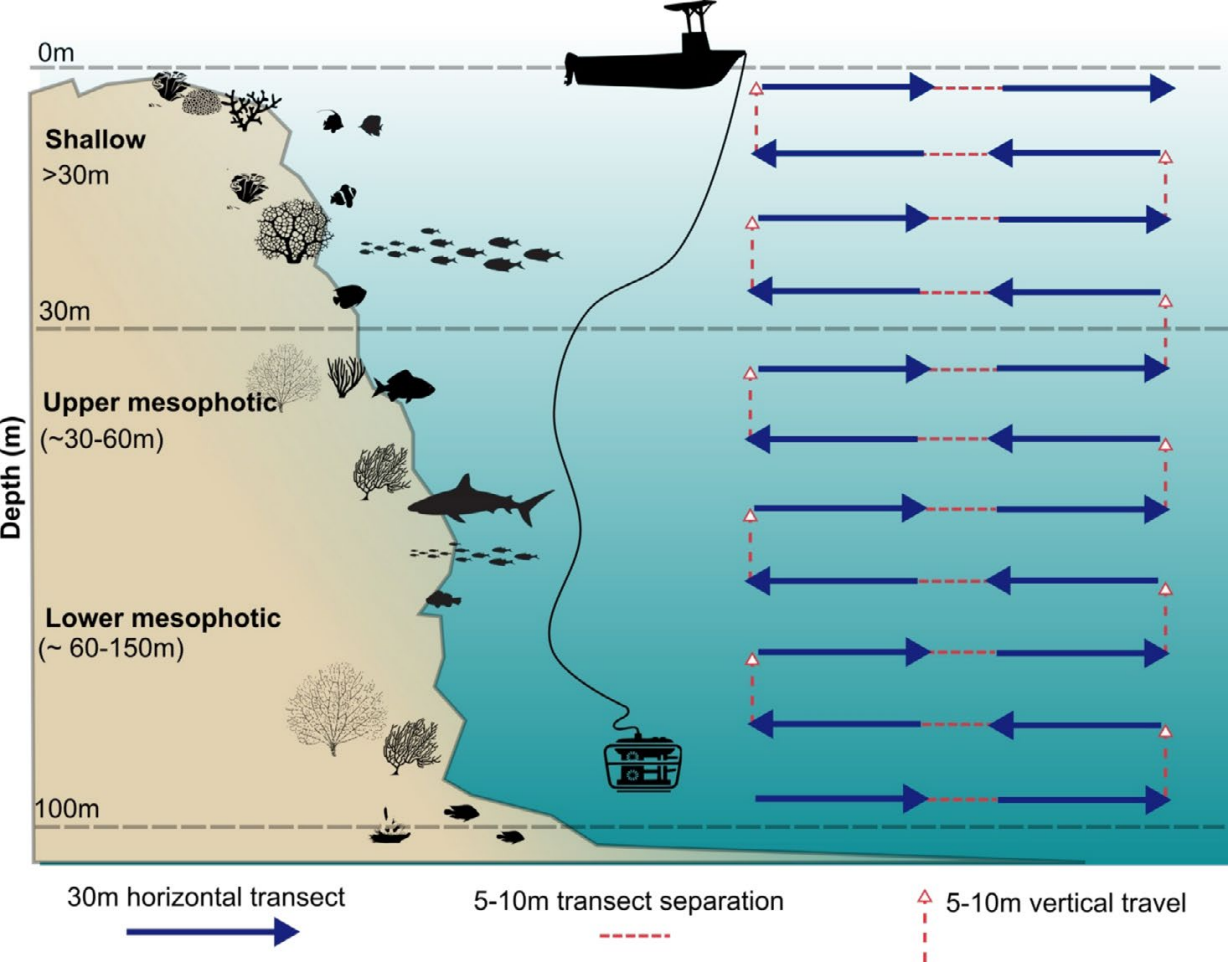
Infographic showing the remotely operated vehicle (ROV) field survey techniques for quantifying fish assemblages within deep reef habitats. Transects were conducted from max 100 m–surface, with 5–10 separation (horizontal and vertical) between transects. Adapted from Galbraith et al. (2022).

Each transect was 150 s in duration and conducted at a known speed (0.2 ms^1^; equating to a transect length of *ca*. 30 m), using the on‐board inertial navigation system, and at a consistent depth (±2 m, via the ROV on‐board depth sensor). Transect start and finish points were indicated on the video by rapidly rotating the ROV left and right, with at least 5 m separating replicate transects at each depth. Once surveys were completed at maximum deployment depth, the ROV ascended at least 10 m to achieve vertical separation, and transects were repeated at the new depth in the same way. This procedure was repeated until the ROV reached the surface, reef crest or the deployment was terminated due to equipment failure or logistical constraints (Figure 2). All data collection was conducted under Great Barrier Reef Marine Park Permit G22/46908.1, Queensland Fisheries Permit 266,351 and JCU Animal Ethics permit A2721.

Videos were subsequently analysed in the software EventMeasure Stereo (SeaGIS Pty Ltd), and all fish counted in a standardised portion of the screen, *ca* 2.5 m either side of the centre of the transect line. Transect area therefore equates to *ca. 150* m^2^.

### 2.3 Data Analysis

From the whole fish assemblage observation data, occurrences of predatory fishes were extracted following protocols outlined in Cresswell et al. (2023). Predator abundance was then calculated as numbers of predatory fish species observed per transect (150m^2^ of reef area). To explore patterns in taxonomic richness, balanced asymptotic species richness estimates were first obtained using sample‐based rarefaction in the R package *iNEXT* (Hsieh et al. 2022), following Chao et al. (2014), in order to obtain balanced asymptotic species richness estimates for each transect. To test the effect of depth on predator abundance and asymptotic species richness, we used hierarchical linear models fitted in *STAN* (Carpenter et al. 2017), via the R package *brms* (Bürkner 2017).

### 2.4 Effect of Depth on Predator Abundance and Species Richness

Separate models were used to investigate the effect of depth on predator abundance and asymptotic species richness, with depth included as a continuous population effect in both models, and site specified as a varying intercept effect. Because depth is collinear with both water temperature and light, these two variables were excluded from model testing. To test for peaks in abundance and species richness among depths, generalised additive models (GAMs) were fitted and assessed against linear models using leave‐one‐out cross‐validation in the package *loo* (Vehtari 2023), where a higher loo score is indicative of a better‐performing model. Model residual diagnostics were conducted in the *DHARMa* package (Hartig 2022); posterior‐predictive checks conducted using the package *bayesplot* (Gabry and Mahr 2024) and sampling chain diagnostics conducted in *rstan* (Stan Development Team 2024).

For the final abundance model, a negative binomial error distribution with a log‐link and zero‐inflation intercept term (to control for null observations in transects) provided the best model structure for the observed data. For the asymptotic species richness model, a hurdle log‐normal distribution with a log‐link provided the best model structure for the data. Estimated marginal effect sizes and contrasts were extracted from posterior model draws using the packages *emmeans* (Lenth 2021) and *tidybayes* (Kay 2023). Effect sizes were extracted from posterior draws as both trends associated with depth and as probability densities, with two thresholds of exceedance probability considered: *p* > 0.8—some evidence in support of an effect; *p* > 0.95 strong—evidence in support of an effect. Contrast estimates were extracted between maximum and minimum depths surveyed and are presented as ratios (minimum depth: maximum depth).

### 2.5 Differences in Assemblage Structure Among Depth Zones

To test for differences in predator assemblage composition with depth, all transects were firstly assigned to one of three categorical depth zones: shallow (0–30 m), upper mesophotic (31–60 m), and lower mesophotic (61–100 m). At the transect level, occurrences of individual predator taxa were low, so analyses of assemblage composition were conducted at both species and family levels, pooled by site and depth zone. Using a multivariate modelling approach, we tested for differences in assemblage composition among depth zones, at the species and family levels, using the *manyglm* function in the R package *mvabund* (Wang et al. 2022). Pairwise differences in assemblages between depths were then identified using likelihood ratio tests (LRT) with the function *anova.manyglm*, which provides multivariate (i.e., combined assemblage) and univariate (i.e., for individual species or families) post hoc comparisons. Univariate *p* value estimates were adjusted for multiple testing using a step‐down resampling procedure, with 999 bootstrap iterations selected. To visualise multivariate differences identified by the *manyglm*, a nonmetric multidimensional scaling (nMDS) was used, while patterns in univariate differences were visualised by extracting taxon abundances from the data, with mean abundance ± S.E. presented.

All data management and graphic production were conducted in the *tidyverse* family of packages (Wickham et al. 2019) in R (R Core Team 2022). Fully reproducible code and model outputs and tables are available at https://github.com/bjcresswell/CoralSeaROVPredsDepth.

## 3 Results

In total, 1037 observations of predator fishes were recorded over 346 ROV video transects (Lihou Reef: 152, Osprey Reef: 130, Bougainville Reef: 64, Figure 1B–D), comprised of 90 transects in the shallows (0–30 m), 125 in the upper‐mesophotic (31–60 m) and 131 in the lower‐mesophotic (> 60 m). A total of 77 different predator species were recorded from 17 families. Linear models outperformed GAMs, with no evidence of depth‐peaks found for either abundance or species richness.

### 3.1 Effect of Depth on Predator Abundance

**FIGURE 3:**
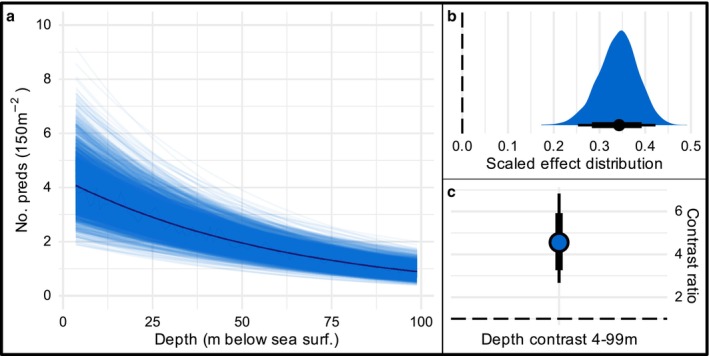
Effect of depth on predatory fish abundance. (a) Modelled trend. Each line represents a posterior draw from the model. (b) Distribution of proportional decline in abundance per 1SD increase in depth. Median decline (34% per 1SD depth) represented black circle. Narrow/wide horizontal bars indicate 95% and 80% area under the curve, respectively. Dashed line at zero represents no effect. (c) Pairwise contrast between the shallowest and deepest depths surveyed in the study (4 and 99 m). Dashed line indicates a 1:1 ratio.

There was strong evidence of an effect of depth on predator abundance (Figure 3a), with numbers of predatory fishes per transect declining by 34% (95% HPDI [25–42]) for every 1 SD of increasing depth (26 m, Figure 3b, *P* = 1). In the shallowest depths surveyed (3.6 m), modelled mean abundance of predators was 4.1 individuals per 150 m^2^ (95% HPDI [2.4–6.0]), while at the greatest survey depths (98.8 m) mean predator abundance was 0.9 individuals per 150 m^2^ (95% HPDI [0.6–1.4]), a greater than 4‐fold decline between the shallowest and deepest depths in the study (4.5:1, 95% HPDI [2.7–6.8], Figure 3c).

### 3.2 Effect of Depth on Predator Species Richness

**FIGURE 4:**
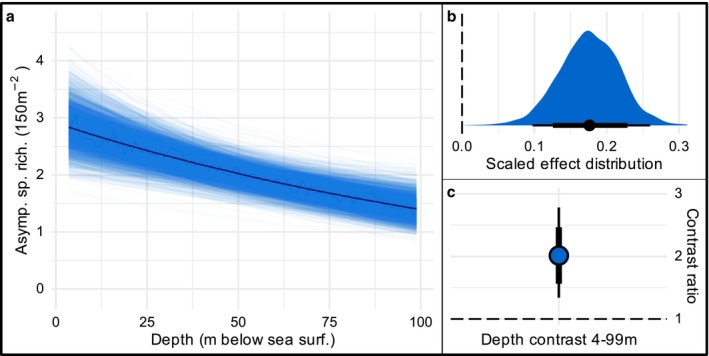
Effect of depth on predatory fish asymptotic species richness. (a) Modelled trend. Each line represents a posterior draw from the model. (b) Distribution of proportional decline in asymptotic species richness per 1SD increase in depth. Median decline (18% per 1SD depth) represented black circle. Narrow/wide horizontal bars indicate 95% and 80% area under the curve, respectively. Dashed line at zero represents no effect. (c) Pairwise contrast between the shallowest and deepest depths surveyed in the study (4 and 99 m). Dashed line indicates a 1:1 ratio.

There was also strong evidence of an effect of depth on asymptotic predator species richness (Figure 4a), with species richness declining by 18% (95% HPDI [10–26]) for every 1 SD of increasing depth (26 m, Figure 4b, *p* = 1). In the shallows, species richness was double that of the deepest surveyed reefs (contrast ratio 2:1, 95% HPDI [1.4–2.7], Figure 4c).

### 3.3 Assemblage Composition

**FIGURE 5:**
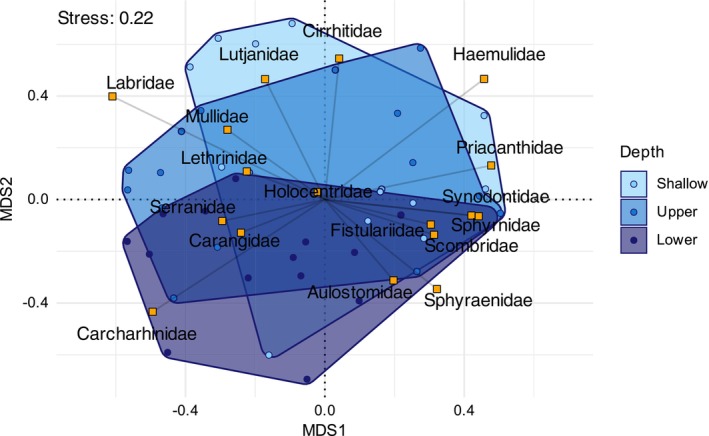
Nonmetric multidimensional scaling of family assemblage composition at each depth zone.

**FIGURE 6:**
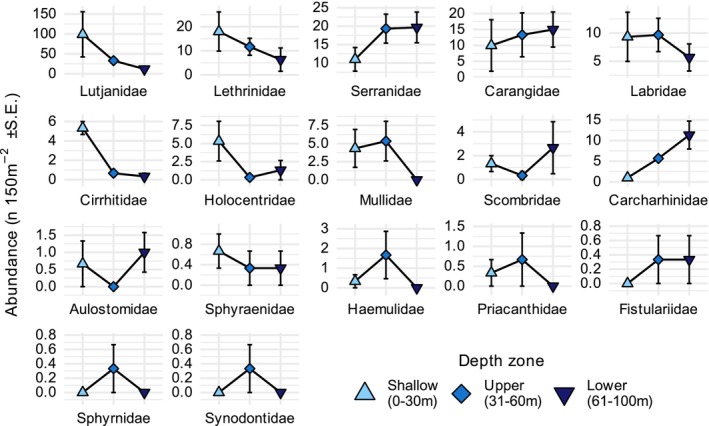
Mean abundance per transect (±S.E.) by depth zone for each of the 17 predatory fish families used in the multivariate model. Panels ordered by most–least abundant families in thestudy.

The multivariate GLMs identified no differences in species assemblage structure among depth zones. However, there were differences identified in family assemblage structure among depth zones (LRT = 75.6, Res.df = 48, *p* = 0.008, Figure 5). Significant post hoc pairwise contrasts were identified between shallow and lower‐mesophotic zones only (Table 1). However, the model identified only one predator family significantly associated with changes in depth, Carcharhinidae (reef sharks), which increased in abundance with depth (LRT 18.09, Adj. *p*: 0.003, Table 2, Figure 6). All other families were nonsignificantly associated with depth‐based differences in assemblage structure.

**TABLE 1:** Pairwise differences in species and family assemblage structure among depths (from manyglm test).

Depth contrast	Family contrast		Species contrast	
	LRT	Adj. *p*	LRT	Adj. *p*
Shallow vs. lower	46.4	0.035	83.6	0.388
Shallow vs. upper	34.6	0.114	64.4	0.523
Upper vs. lower	33.1	0.144	111.2	0.173

*Note:* *p* values adjusted for multiple comparisons are presented.

Abbreviation: LRT, likelihood ratio test.

**TABLE 2:** Statistical significance of family abundance to pairwise differences in assemblage structure.

Family	Common name	LRT	Adj. *p*	Family	Common name	LRT	Adj. *p*
Carcharhinidae	Reef sharks	18.09	0.003*	Sphyrnidae	Hammerhead sharks	2.2	0.984
Cirrhitidae	Hawkfishes	10.8	0.075	Scombridae	Tunas	2.12	0.984
Mullidae	Goatfishes	8.56	0.207	Priacanthidae	Big eyes	1.85	0.984
Lutjanidae	Snappers	7.72	0.259	Serranidae	Groupers	1.79	0.984
Haemulidae	Emperors	6.97	0.291	Fistulariidae	Cornetfishes	1.62	0.984
Aulostomidae	Trumpetfishes	4.26	0.827	Labridae	Wrasses	0.58	0.984
Holocentridae	Soldierfishes	3.4	0.891	Sphyraenidae	Barracudas	0.47	0.984
Lethrinidae	Emperors	2.78	0.928	Carangidae	Trevallies	0.24	0.984
Synodontidae	Lizardfishes	2.2	0.984				

## 4 Discussion

Declines in assemblage species richness and abundance with depth are well documented for coral reef fishes across the tropics (Pinheiro, MacDonald, Quimbayo, et al. 2023), including on shallow reefs, and increasingly over mesophotic depth ranges (Sih et al. 2017; Brown et al. 2022). Predatory fish assemblages on seamount reefs of the Coral Sea exhibited the same depth‐associated changes, with decreases in both abundance and species richness from the surface down to ~100 m. Shallow waters supported higher abundance (4.5:1) and species richness (2.4:1) than the deepest depths surveyed. Previous studies have suggested three dominant drivers of declines in coral reef fishes with depth: declining benthic complexity, diminishing food sources, and changes in hydrodynamic regimes. These drivers are likely interconnected and rarely operate in isolation. The generally described pattern of decreasing benthic complexity with depth reduces the availability and diversity of habitats and/or shelter for both prey fishes (Gratwicke and Speight 2005) and certain reef predators (Wen et al. 2013). This driver might be inhibited in the Coral Sea because clear, oligotrophic water allows penetration of light to substantial depths and consequently there are substantial tracts of complex coral habitats at mesophotic depths in the region (Bridge et al. 2011; Galbraith et al. 2024). Indeed, a shift from photosynthetic corals to azooxanthellate filter feeders has been reported to occur as deep as 60 m in the Coral Sea (Dalzell et al. 1996). Benthic complexity was not directly estimated for each transect in the present study, and future iterations of this work could include per‐transect estimates or measurements of benthic complexity and configuration.

The presence of energy gradients with depth can gradually limit species richness as energy decreases (species‐energy hypothesis, Wright 1983). The explanatory power of this driver is appealing, given the proposed universality of species‐energy theory. In addition, on coral reefs, this effect is straightforward to conceptualise: as light diminishes, so does the abundance of photosynthetic, habitat‐forming organisms, and models exist to explain benthic community transitions along depth‐associated light attenuation (Laverick et al. 2020). The result of this is resource limitation for the herbivorous, corallivorous and omnivorous fishes that depend on them, and consequent declines in numbers in these trophic guilds with increasing depth (Beukers and Jones 1998). Declines in habitat complexity and primary food sources with depth have obvious implications for herbivores and other lower trophic guilds, but are less likely to apply directly to predatory fishes, except through second‐order pathways, by limiting prey availability at depth (Beukers‐Stewart et al. 2011). Further research is required to directly assess the impact of prey availability across the depth gradient on predatory species.

A third possible explanatory driver is hydrodynamics, which may be directly relevant to the predator assemblage. Wave action, currents and other water movements have structural effects on reef fish assemblages (McGehee 1994; Bellwood et al. 2002; Depczynski and Bellwood 2005; Lamb et al. 2020; Galbraith et al. 2023), and hydrodynamic regimes will play a role in structuring fish assemblages on seamounts in this study, but almost certainly in a different way to how these forces operate on continuous coastal reefs. The distinct and powerful hydrodynamic forces that occur on seamounts have been linked to the aggregation of predator fishes on these structures (Morato et al. 2010; Fontes et al. 2014; Letessier et al. 2019), likely through mechanisms that sustain a wide variety of prey species, such as planktivores. Plankton tows and the quantification of hydrodynamic variables (e.g., upwelling) among depths were unfeasible within this study. However, future research on the systems in this study and on seamount coral reefs in general could include measures of both the underlying hydrodynamics as well as plankton community structure as predictors of predator assemblages across mesophotic depths.

The presence of an electronic, tethered survey vehicle, such as the BlueROV2 used in this study, has the potential to influence fish behaviour. While we are not aware of peer‐reviewed studies that assess fish responses specifically to the tether of small, micro‐ROVs in shallow reef environments, recent work has been directed at assessing comparative sampling between SCUBA diver visual census methods, SCUBA diver video methods, and ROV video methods. Mostly, these have found that while fish behaviour does indeed differ towards divers conducting visual counts and ROVs (Jessop et al. 2024), results are similar between video methods conducted by divers and by ROVs (Schramm et al. 2020; Jessop et al. 2022). Interestingly, one study (Hellmrich et al. 2023) found that fish allowed ROVs to approach closer than SCUBA diver operated videos (DOV) suggesting that any adverse behavioural response was lower for the ROV compared to diver‐based surveys. Variation in behavioural traits like flight initiation distance (FID) might contribute to our ability to detect depth‐related differences in assemblage structure, and future work could assess the behavioural responses of reef fish to tethered ROVs under varying conditions and across species.

In the present study, declines in seamount predator species richness and abundance were monotonic. The absence of a peak in either species richness or abundance at intermediate depths is in keeping with a number of studies of mesophotic reef fish assemblages (Lukens 1981; Pinheiro, MacDonald, Quimbayo, et al. 2023); however, fish assemblages can possess peaks at mesophotic depths (Brokovich et al. 2008; Hoban et al. 2023) for at least two reasons. The first is that planktivore abundance often increases with depth on reef slopes, reaching a point where this guild can comprise almost 100% of an assemblage at mesophotic depths (Thresher and Colin 1986; Pereira‐Filho et al. 2011). While not directly relevant to the predator assemblage, the resulting increase in abundance and variety of planktivorous prey could support high abundance of predators at these depths. The second potential reason for mesophotic peaks in richness is the co‐occurrence of mesophotic specialists with shallow and deep‐water species at the lower and upper extremes of their depth ranges, respectively. Rocha et al. (2018) found that 27% of shallow reef fishes were also found in the mesophotic zone, despite overall high levels of dissimilarity between depth zones. For predatory taxa, these patterns seem conceptually plausible; however, they were either absent or not detected for the organisms in this study. The mobile characteristic of many predator species may moderate delineations in habitat preference, particularly those recorded using instantaneous survey methods including ROV and in situ UVC, making peaks in diversity and abundance difficult to detect. Coleman et al. (2018) highlight how declines in diversity with depth in Micronesia could potentially be attributed to lower sample sizes at depth; however, for this study, the largest number of transects was conducted in the two deepest zones. Tagging and tracking depth movements for a number of species over time would be an avenue for quantifying depth preferences and specialism in predator species.

While declines in abundance and richness across the depth gradient were as predicted, the lack of differences in assemblage composition was not expected. The only difference detected by the multivariate models was in family composition between the shallows and the lower mesophotic. This is a potentially surprising result because a wide variety of organisms occurring in the mesophotic are restricted to those depths (Colin 1974; Thresher and Colin 1986; Rosa et al. 2016). This phenomenon may not apply to mobile predators; however, research from across the Pacific has demonstrated general faunal shifts in fish species assemblages corresponding to major thermoclines (Pyle et al. 2019). Thermocline depths vary globally as a result of variability in local and regional hydrodynamics (Pomar et al. 2012; Pyle et al. 2019), and this variation is likely to be associated with corresponding peaks in species richness or breaks in assemblage structure. Such abrupt changes in temperature are likely absent or not pronounced for the seamounts of the Coral Sea (Ceccarelli et al. 2013; Brooke and Schmidt Ocean Institute 2022). There is, however, a known seasonal variability in SST of around 5°C, a pattern which attenuates at approximately 100 m depth. Predator taxa with small thermal tolerances would be expected to converge on deeper depths or adjust their mean depth occupancy, utilising gradients in water temperature to mitigate raised SSTs. At present, no studies have examined the potential for this behaviour in a seamount predator.

The only taxon significantly associated with assemblage differences among depth zones was Carcharhinidae, which were over 10× more abundant in the lower‐mesophotic than the shallows. This was unexpected, as these organisms are probably some of the most mobile reef‐associated predators and are known to be abundant in the shallows on at least one of the seamounts in this study (Osprey Reef: Barnett et al. 2012), although some reef sharks have been shown to derive food from MCEs (Papastamatiou et al. 2015). There were a number of families with noteworthy differences in abundance among depth zones, but these were not significantly associated with differences in the predator assemblage among depth zones. Patterns for these families included increases in abundance with depth (e.g., Serranidae, Carangidae) and also peaks in the upper mesophotic (e.g., Haemulidae, Priacanthidae). Most of these families were rare (seven families had low single‐digit abundances per transect across the entire study), which potentially explains the lack of contribution to overall assemblage differences, but suggests that some of these taxa might be associated with particular depths and vulnerable to depth‐specific stressors. One family that is dominant on mesophotic reefs is Serranidae (Pyle et al. 2019) and was among the most abundant families found at depth in this study. Most studies describing the abundance of this family at depth normally combine both the piscivorous (e.g., *Epinephelus*, *Plectropomus* spp.) and the planktivorous serranids (e.g., *Pseudanthias* spp.) (Thresher and Colin 1986; Brokovich et al. 2008; Sih et al. 2017; Pinheiro, MacDonald, Quimbayo, et al. 2023). Planktivorous serranids were obviously excluded from this study, so the abundance of the piscivorous component suggests that depth preferences in this family may have evolutionary as well as ecological origins.

It is suggested that depth can act as a refuge for shallow water species during periods of environmental stress (deep reef refugia hypothesis (Hughes and Tanner 2000; Riegl and Piller 2003; Bongaerts et al. 2010)). The hypothesis is built on two main underlying assumptions: (i) that there is overlap in species composition between depth zones and (ii) that deeper reefs are less susceptible to anthropogenic impacts than shallow reefs (Rocha et al. 2018). Results presented here suggest that deeper water is probably usable for a range of seamount reef predators, but that there may be general declines in abundance of these species with depth. Even for those taxa capable of using broad depth ranges, there may be impacts of greater depth use on body condition (e.g., growth, fecundity) (Hoey et al. 2007) or trophic position (Bradley et al. 2016), and this is a promising avenue of investigation for predators at the top of the food web. Depth does not represent a universal refuge for predatory fishes on seamount reefs, in keeping with similar observations for other taxa (Bongaerts et al. 2017). A further related climate‐driven phenomenon that is likely to occur over the next century is vertical migration of surface isotherms, which are predicted to deepen at a rate of up to 32.3 m dec^−1^ under a business‐as‐usual scenario (Jorda et al. 2020). If this situation materialises, it will compress the amount of vertical habitat available to all occupants of coral reefs. Whether predators on seamount reefs such as those of the Coral Sea will be able to adapt or acclimate to such scenarios is unknown. Furthermore, whether deeper reefs or isolated seamounts are actually protected from human impacts is also still uncertain. Recent research has found evidence of plastics and other pollution on some of the world's deepest and most isolated seamount and atoll reefs (Pinheiro, MacDonald, Santos, et al. 2023), including those in this study, despite all three seamounts in this study almost entirely falling inside a protected area.

Overall, the seamount reefs in this study support abundant and diverse assemblages of predatory fishes, largely protected from fishing pressure by incorporation into well‐defined, no‐take marine protected areas. A large number of taxa can function across the wide range of depths that are available, offering some hope that depth can operate as a refuge for predatory fish families on these structures, which may be important given the likely loss of shallow reef habitat under future climate scenarios. However, further research is required to understand individual species responses to changing surface water temperatures and whether these can be used to mitigate the effect of warmer conditions on internal body temperatures.

## Author Contributions

**B. J. Cresswell:** conceptualization (lead), data curation (lead), formal analysis (lead), funding acquisition (supporting), investigation (equal), methodology (equal), project administration (lead), resources (equal), software (lead), validation (equal), visualization (equal), writing – original draft (lead), writing – review and editing (lead). **G. F. Galbraith:** conceptualization (equal), data curation (equal), formal analysis (equal), investigation (equal), methodology (equal), project administration (equal), validation (equal), visualization (equal), writing – review and editing (equal). **A. Barnett:** funding acquisition (equal), investigation (equal), resources (equal), supervision (equal), writing – review and editing (equal). **H. B. Harrison:** conceptualization (equal), formal analysis (supporting), investigation (supporting), supervision (equal), writing – review and editing (equal). **G. P. Jones:** conceptualization (equal), formal analysis (equal), investigation (equal), supervision (equal), writing – original draft (equal). **E. C. McClure:** data curation (equal), formal analysis (supporting), investigation (equal), project administration (equal), writing – review and editing (equal). **T. J. R. Quimpo:** data curation (equal), investigation (equal), project administration (equal), writing – review and editing (equal). **A. S. Hoey:** formal analysis (supporting), funding acquisition (equal), investigation (equal), resources (equal), supervision (equal), writing – review and editing (equal).

## Conflicts of Interest

The authors declare no conflicts of interest.

## Data Availability

Data and code for the analyses in this study can be accessed at: https://github.com/bjcresswell/CoralSeaROVPredsDepth.

## References

[ece372044-bib-0001] Andradi‐Brown, D. A. , E. Gress , G. Wright , D. A. Exton , and A. D. Rogers . 2016. “Reef Fish Community Biomass and Trophic Structure Changes Across Shallow to Upper‐Mesophotic Reefs in the Mesoamerican Barrier Reef, Caribbean.” PLoS One 11, no. 6: 1–19. 10.1371/journal.pone.0156641. PMC4917088 27332811

[ece372044-bib-0002] Baletaud, F. , G. Lecellier , A. Gilbert , et al. 2023. “Comparing Seamounts and Coral Reefs With eDNA and BRUVS Reveals Oases and Refuges on Shallow Seamounts.” Biology 12, no. 11: 1446. 10.3390/biology12111446. 37998045 PMC10669620

[ece372044-bib-0003] Barnett, A. , K. G. Abrantes , J. Seymour , and R. Fitzpatrick . 2012. “Residency and Spatial Use by Reef Sharks of an Isolated Seamount and Its Implications for Conservation.” PLoS One 7, no. 5: e36574. 10.1371/journal.pone.0036574. 22615782 PMC3353940

[ece372044-bib-0004] Bellwood, D. R. , P. C. Wainwright , C. J. Fulton , and A. Hoey . 2002. “Assembly Rules and Functional Groups at Global Biogeographical Scales.” Functional Ecology 16, no. 5: 557–562. https://www.jstor.org/stable/826738.

[ece372044-bib-0005] Beukers, J. S. , and G. P. Jones . 1998. “Habitat Complexity Modifies the Impact of Piscivores on a Coral Reef Fish Population.” Oecologia 114, no. 1: 50–59. 10.1007/s004420050419. 28307557

[ece372044-bib-0006] Beukers‐Stewart, B. D. , J. S. Beukers‐Stewart , and G. P. Jones . 2011. “Behavioural and Developmental Responses of Predatory Coral Reef Fish to Variation in the Abundance of Prey.” Coral Reefs 30, no. 3: 855–864. 10.1007/s00338-011-0792-9.

[ece372044-bib-0007] Bongaerts, P. 2022. “Mesophotic Coral Ecosystems.” Current Biology 32, no. 8: R345–R346. 10.1016/j.cub.2022.03.036. 35472416

[ece372044-bib-0008] Bongaerts, P. , T. Ridgway , E. M. Sampayo , and O. Hoegh‐Guldberg . 2010. “Assessing the “Deep Reef Refugia” Hypothesis: Focus on Caribbean Reefs.” Coral Reefs 29, no. 2: 1–19. 10.1007/s00338-009-0581-x.

[ece372044-bib-0009] Bongaerts, P. , C. Riginos , R. Brunner , N. Englebert , S. R. Smith , and O. Hoegh‐Guldberg . 2017. “Deep Reefs Are Not Universal Refuges: Reseeding Potential Varies Among Coral Species.” Science Advances 3, no. 2: e1602373. 10.1126/sciadv.1602373. 28246645 PMC5310828

[ece372044-bib-0010] Bradley, C. J. , K. Longenecker , R. L. Pyle , and B. N. Popp . 2016. “Compound‐Specific Isotopic Analysis of Amino Acids Reveals Dietary Changes in Mesophotic Coral‐Reef Fish.” Marine Ecology Progress Series 558 Glynn 1996: 65–79. 10.3354/meps11872.

[ece372044-bib-0011] Bridge, T. C. L. , T. J. Done , A. Friedman , et al. 2011. “Variability in Mesophotic Coral Reef Communities Along the Great Barrier Reef, Australia.” Marine Ecology Progress Series 428: 63–75. 10.3354/meps09046.

[ece372044-bib-0012] Bridges, A. E. H. , D. K. A. Barnes , J. B. Bell , R. E. Ross , and K. L. Howell . 2022. “Depth and Latitudinal Gradients of Diversity in Seamount Benthic Communities.” Journal of Biogeography 49: 904–915. 10.1111/jbi.14355.

[ece372044-bib-0013] Brokovich, E. , S. Einbinder , N. Shashar , M. Kiflawi , and S. Kark . 2008. “Descending to the Twilight‐Zone: Changes in Coral Reef Fish Assemblages Along a Depth Gradient Down to 65 m.” Marine Ecology Progress Series 371: 253–262. 10.3354/meps07591.

[ece372044-bib-0014] Brooke, B. , and Schmidt Ocean Institute . 2022. “Schmidt Ocean Institute Expedition Report: Seamounts, Canyons and Reefs of the Coral Sea.” *Zenodo*. 10.5281/zenodo.7308219.

[ece372044-bib-0015] Brown, K. , J. Monk , J. Williams , A. Carroll , D. Harasti , and N. Barrett . 2022. “Depth and Benthic Habitat Influence Shallow and Mesophotic Predatory Fishes on a Remote, High‐Latitude Coral Reef.” PLoS One 17, no. 3: e0265067. 10.1371/journal.pone.0265067. 35324946 PMC8947262

[ece372044-bib-0016] Bürkner, P.‐C. 2017. “brms: An R Package for Bayesian Multilevel Models Using Stan.” Journal of Statistical Software 80, no. 1: 1–28. 10.18637/jss.v080.i01.

[ece372044-bib-0017] Carpenter, B. , A. Gelman , M. D. Hoffman , et al. 2017. “Stan: A Probabilistic Programming Language.” Journal of Statistical Software 76: 1–31. 36568334 10.18637/jss.v076.i01 PMC9788645

[ece372044-bib-0018] Carr, M. H. , T. W. Anderson , and M. A. Hixon . 2002. “Biodiversity, Population Regulation, and the Stability of Coral‐Reef Fish Communities.” Proceedings of the National Academy of Sciences of the United States of America 99, no. 17: 11241–11245. 10.1073/pnas.162653499. 12177430 PMC123240

[ece372044-bib-0019] Ceccarelli, D. M. , A. D. McKinnon , S. Andréfouët , et al. 2013. “The Coral Sea: Physical Environment, Ecosystem Status and Biodiversity Assets.” Advances in Marine Biology 66: 213–290. 10.1016/B978-0-12-408096-6.00004-3. 24182902

[ece372044-bib-0020] Chao, A. , N. J. Gotelli , T. C. Hsieh , et al. 2014. “Rarefaction and Extrapolation With Hill Numbers: A Framework for Sampling and Estimation in Species Diversity Studies the Harvard Community Has Made This Article Openly Available. Please Share How This Access Benefits You. Your Story Matters.” Ecological Monographs 84, no. 1: 45–67.

[ece372044-bib-0021] Coleman, R. R. , J. M. Copus , D. M. Coffey , R. K. Whitton , and B. W. Bowen . 2018. “Shifting Reef Fish Assemblages Along a Depth Gradient in Pohnpei, Micronesia.” PeerJ 6: e4650. 10.7717/peerj.4650. 29707432 PMC5922234

[ece372044-bib-0022] Colin, P. L. 1974. “Observation and Collection of Deep‐Reef Fishes Off the Coasts of Jamaica and British Honduras (Belize).” Marine Biology 24, no. 1: 29–38. 10.1007/BF00402844.

[ece372044-bib-0023] Cooper, A. M. , C. MacDonald , T. E. Roberts , and T. C. L. Bridge . 2019. “Variability in the Functional Composition of Coral Reef Fish Communities on Submerged and Emergent Reefs in the Central Great Barrier Reef, Australia.” PLoS One 14, no. 5: e0216785. 10.1371/journal.pone.0216785. 31100087 PMC6524821

[ece372044-bib-0024] Costello, M. J. , and C. Chaudhary . 2017. “Marine Biodiversity, Biogeography, Deep‐Sea Gradients, and Conservation.” Current Biology 27, no. 11: R511–R527. 10.1016/j.cub.2017.04.060. 28586689

[ece372044-bib-0025] Cresswell, B. J. , G. F. Galbraith , H. B. Harrison , M. McCormick , and G. P. Jones . 2023. “Coral Reef Pinnacles Act as Ecological Magnets for the Abundance, Diversity and Biomass of Predatory Fishes.” Marine Ecology Progress Series 717: 143–156. 10.3354/meps14377.

[ece372044-bib-0026] Dalzell, P. , T. J. H. Adams , and N. V. C. Polunin . 1996. “Coastal Fisheries in the Pacific Islands.” Oceanography and Marine Biology: An Annual Review 34: 395–531.

[ece372044-bib-0027] Depczynski, M. , and D. R. Bellwood . 2005. “Wave Energy and Spatial Variability in Community Structure of Small Cryptic Coral Reef Fishes.” Marine Ecology Progress Series 303: 283–293. 10.3354/meps303283.

[ece372044-bib-0028] Done, T. J. 1982a. “Coral Zonation: Its Nature and Significance’.” In Perspectives on Coral Reefs, edited by D. Barnes and B. Clouston , 107–147. Brian Cloustin Publisher. https://cir.nii.ac.jp/crid/1570009749212998656.bib?lang=en.

[ece372044-bib-0029] Done, T. J. 1982b. “Patterns in the Distribution of Coral Communities Across the Central Great Barrier Reef.” Coral Reefs 1, no. 2: 95–107. 10.1007/BF00301691.

[ece372044-bib-0030] Fischer, A. G. 1960. “Latitudinal Variations in Organic Diversity.” Evolution 14, no. 1: 64–81.

[ece372044-bib-0031] Fontes, J. , M. Schmiing , and P. Afonso . 2014. “Permanent Aggregations of a Pelagic Predator at Shallow Seamounts.” Marine Biology 161, no. 6: 1349–1360. 10.1007/s00227-014-2423-9.

[ece372044-bib-0032] Fukunaga, A. , R. K. Kosaki , D. Wagner , and C. Kane . 2016. “Structure of Mesophotic Reef Fish Assemblages in the Northwestern Hawaiian Islands.” PLoS One 11, no. 7: 1–15. 10.1371/journal.pone.0157861. PMC4934686 27383614

[ece372044-bib-0033] Gabry, J. , and T. Mahr . 2024. “bayesplot: Plotting for Bayesian Models.” R Package Version 1.11.1.

[ece372044-bib-0034] Galbraith, G. , E. McClure , and A. Barnett . 2022. “Diving Into the Deep: The Unique Deep Habitats of the Coral Sea Marine Park.” Report prepared for Parks Australia. 10.13140/RG.2.2.10516.68487/1.

[ece372044-bib-0035] Galbraith, G. F. , B. J. Cresswell , E. C. McClure , and A. S. Hoey . 2024. “Tropical Seamounts as Stepping‐Stones for Coral Reef Fishes: Range Extensions and New Regional Distributions From Mesophotic Ecosystems in the Coral Sea, Australia.” Marine Biodiversity 54: 17. 10.1007/s12526-024-01404-0.

[ece372044-bib-0036] Galbraith, G. F. , B. J. Cresswell , M. I. McCormick , T. C. Bridge , and G. P. Jones . 2021. “High Diversity, Abundance and Distinct Fish Assemblages on Submerged Coral Reef Pinnacles Compared to Shallow Emergent Reefs.” Coral Reefs 40: 335–354. 10.1007/s00338-020-02044-z.

[ece372044-bib-0037] Galbraith, G. F. , B. J. Cresswell , M. I. McCormick , and G. P. Jones . 2023. “Strong Hydrodynamic Drivers of Coral Reef Fish Biodiversity on Submerged Pinnacle Coral Reefs.” Limnology and Oceanography 9999: 1–16. 10.1002/lno.12431.

[ece372044-bib-0038] Genin, A. 2004. “Bio‐Physical Coupling in the Formation of Zooplankton and Fish Aggregations Over Abrupt Topographies.” Journal of Marine Systems 50: 3–20. 10.1016/j.jmarsys.2003.10.008.

[ece372044-bib-0039] Genin, A. , and J. F. Dower . 2007. “Seamount Plankton Dynamics.” In Seamounts: Ecology, Fisheries & Conservation, edited by T. J. Pitcher , T. Morato , P. J. B. Hart , M. R. Clark , N. Haggan , and R. S. Santos , 85–100. Blackwell Publishing.

[ece372044-bib-0040] Goetze, J. S. , T. Bond , D. L. McLean , et al. 2019. “A Field and Video Analysis Guide for Diver Operated Stereo‐Video.” Methods in Ecology and Evolution 10, no. 7: 1083–1090. 10.1111/2041-210X.13189.

[ece372044-bib-0041] González‐Sansón, G. , C. Aguilar , I. Hernández , and Y. Cabrera . 2009. “Effects of Depth and Bottom Communities on the Distribution of Highly Territorial Reef Fish in the Northwestern Region of Cuba.” Journal of Applied Ichthyology 25, no. 6: 652–660. 10.1111/j.1439-0426.2009.01332.x.

[ece372044-bib-0042] Gratwicke, B. , and M. R. Speight . 2005. “The Relationship Between Fish Species Richness, Abundance and Habitat Complexity in a Range of Shallow Tropical Marine Habitats.” Journal of Fish Biology 66, no. 3: 650–667. 10.1111/j.0022-1112.2005.00629.x.

[ece372044-bib-0043] Gutiérrez, L. 1998. “Habitat Selection by Recruits Establishes Local Patterns of Adult Distribution in Two Species of Damselfishes: Stegastes Dorsopunicans and *S. planifrons* .” Oecologia 115, no. 1–2: 268–277. 10.1007/s004420050516. 28308462

[ece372044-bib-0044] Halpern, B. S. , S. Walbridge , K. A. Selkoe , et al. 2008. “A Global Map of Human Impact on Marine Ecosystems.” Science 319, no. 5865: 948–952. 10.1126/science.1149345. 18276889

[ece372044-bib-0045] Hamner, W. M. , M. S. Jones , J. H. Carleton , I. R. Hauri , and D. M. Williams . 1988. “Zooplankton, Planktivorous Fish, and Water Currents on a Windward Reef Face: Great Barrier Reef, Australia.” Bulletin of Marine Science 42, no. 3: 459–479.

[ece372044-bib-0046] Hartig, F. 2022. “DHARMa: Residual Diagnostics for Hierarchical (Multi‐Level/Mixed) Regression Models.” R Package Version 0.4.5. https://cran.r‐project.org/package=DHARMa.

[ece372044-bib-0047] Hellmrich, L. S. , B. J. Saunders , J. R. C. Parker , J. S. Goetze , and E. S. Harvey . 2023. “Stereo‐ROV Surveys of Tropical Reef Fishes Are Comparable to Stereo‐DOVs With Reduced Behavioural Biases.” Estuarine, Coastal and Shelf Science 281: 108210. 10.1016/j.ecss.2022.108210.

[ece372044-bib-0048] Hinderstein, L. M. , J. C. A. Marr , F. A. Martinez , et al. 2010. “Theme Section on “Mesophotic Coral Ecosystems: Characterization, Ecology, and Management”.” Coral Reefs 29, no. 2: 247–251. 10.1007/s00338-010-0614-5.

[ece372044-bib-0049] Hixon, M. , and G. P. Jones . 2005. “Competition, Predation, and Density‐Dependent Mortality in Demersal Marine Fishes.” Ecology 86, no. 11: 2847–2859. 10.1890/03-8024.

[ece372044-bib-0050] Hixon, M. A. 1991. “Predation as a Process Structuring Coral Reef Fish Communities.” In The Ecology of Fishes on Coral Reefs, 475–508. Academic Press. 10.1016/b978-0-08-092551-6.50022-2.

[ece372044-bib-0051] Hixon, M. A. 2015. “Predation: Piscivory and the Ecology of Coral Reef Fishes.” In Ecology of Fishes on Coral Reefs, edited by C. Mora , 41–52. Cambridge University Press.

[ece372044-bib-0052] Hixon, M. A. , and M. S. Webster . 2002. “Density Dependence in Marine Fishes: Coral‐Reef Populations as Model Systems.” In Coral Reef Fishes: Dynamics and Diversity in a Complex Ecosystem, 303–325. Academic Press.

[ece372044-bib-0053] Hoban, M. L. , M. Bunce , and B. W. Bowen . 2023. “Plumbing the Depths With Environmental DNA (eDNA): Metabarcoding Reveals Biodiversity Zonation at 45 – 60 m on Mesophotic Coral Reefs.” Molecular Ecology 00: 1–19. 10.1111/mec.17140. 37728237

[ece372044-bib-0054] Hoey, J. , M. I. McCormick , and A. S. Hoey . 2007. “Influence of Depth on Sex‐Specific Energy Allocation Patterns in a Tropical Reef Fish.” Coral Reefs 26, no. 3: 603–613. 10.1007/s00338-007-0246-6.

[ece372044-bib-0055] Hsieh, T. C. , K. H. Ma , and A. Chao . 2022. “iNEXT: iNterpolation and EXTrapolation for Species Diversity.” R Package Version 3.0.0. http://chao.stat.nthu.edu.tw/wordpress/software‐download/.

[ece372044-bib-0056] Hughes, T. P. , and J. E. Tanner . 2000. “Recruitment Failure, Life Histories, and Long‐Term Decline of Caribbean Corals.” Ecology 81, no. 8: 2250–2263. 10.1890/0012-9658(2000)081[2250:RFLHAL]2.0.CO;2.

[ece372044-bib-0057] Jankowski, M. W. , N. A. J. Graham , and G. P. Jones . 2015. “Depth Gradients in Diversity, Distribution and Habitat Specialisation in Coral Reef Fishes: Implications for the Depth‐Refuge Hypothesis.” Marine Ecology Progress Series 540: 203–215. 10.3354/meps11523.

[ece372044-bib-0058] Jessop, S. A. , B. J. Saunders , J. S. Goetze , N. S. Barrett , and E. S. Harvey . 2024. “A Comparison of the Behavioural Responses of Fishes to a Remotely Operated Vehicle and Diver‐Based Stereo‐Video Sampling.” Estuarine, Coastal and Shelf Science 298, no. January: 108621. 10.1016/j.ecss.2024.108621.

[ece372044-bib-0059] Jessop, S. A. , B. J. Saunders , J. S. Goetze , and E. S. Harvey . 2022. “A Comparison of Underwater Visual Census, Baited, Diver Operated and Remotely Operated Stereo‐Video for Sampling Shallow Water Reef Fishes.” Estuarine, Coastal and Shelf Science 276, no. July: 108017. 10.1016/j.ecss.2022.108017.

[ece372044-bib-0060] Jorda, G. , N. Marbà , S. Bennett , J. Santana‐Garcon , S. Agusti , and C. M. Duarte . 2020. “Ocean Warming Compresses the Three‐Dimensional Habitat of Marine Life.” Nature Ecology & Evolution 4, no. 1: 109–114. 10.1038/s41559-019-1058-0. 31900450

[ece372044-bib-0061] Kay, M. 2023. “tidybayes: Tidy Data and Geoms for Bayesian Models.” R Package Version 3.0.4. 10.5281/zenodo.1308151.

[ece372044-bib-0062] Khalaf, M. A. , and M. Kochzius . 2002. “Community Structure and Biogeography of Shore Fishes in the Gulf of Aqaba, Red Sea.” Helgoland Marine Research 55, no. 4: 252–284. 10.1007/s10152-001-0090-y.

[ece372044-bib-0063] Lamb, R. W. , F. Smith , and J. D. Witman . 2020. “Consumer Mobility Predicts Impacts of Herbivory Across an Environmental Stress Gradient.” Ecology 101, no. 1: 1–17. 10.1002/ecy.2910. 31605535

[ece372044-bib-0064] Laverick, J. H. , R. Tamir , G. Eyal , and Y. Loya . 2020. “A Generalized Light‐Driven Model of Community Transitions Along Coral Reef Depth Gradients.” Global Ecology and Biogeography 29, no. 9: 1554–1564. 10.1111/geb.13140.

[ece372044-bib-0065] Lecchini, D. , M. Adjeroud , M. S. Pratchett , L. Cadoret , and R. Galzin . 2003. “Spatial Structure of Coral Reef Fish Communities in the Ryukyu Islands, Southern Japan.” Oceanologica Acta 26, no. 5–6: 537–547. 10.1016/S0399-1784(03)00048-3.

[ece372044-bib-0066] Leis, J. M. 1986. “Vertical and Horizontal Distribution of Fish Larvae Near Coral Reefs at Lizard Island, Great Barrier Reef.” Marine Biology 90, no. 4: 505–516. 10.1007/BF00409271.

[ece372044-bib-0067] Lenth, R. 2021. “Emmeans: Estimated Marginal Means, Aka Least‐Squares Means.” R Package [Preprint]. 10.1080/00031305.1980.10483031.

[ece372044-bib-0068] Letessier, T. B. , M. J. Cox , J. J. Meeuwig , P. H. Boersch‐Supan , and A. S. Brierley . 2016. “Enhanced Pelagic Biomass Around Coral Atolls.” Marine Ecology Progress Series 546: 271–276. 10.3354/meps11675.

[ece372044-bib-0069] Letessier, T. B. , D. Mouillot , P. J. Bouchet , et al. 2019. “Remote Reefs and Seamounts Are the Last Refuges for Marine Predators Across the IndoPacific.” PLoS Biology 17, no. 8: e3000366. 10.1371/journal.pbio.3000366. 31386657 PMC6684043

[ece372044-bib-0070] Lukens, R. R. 1981. “Ichthyofaunal Colonization of a New Artificial Reef in the Northern Gulf of Mexico.” Gulf Research Reports 7, no. 1: 41–46. 10.18785/grr.0701.06.

[ece372044-bib-0071] MacDonald, C. , T. C. L. Bridge , and G. P. Jones . 2016. “Depth, Bay Position and Habitat Structure as Determinants of Coral Reef Fish Distributions: Are Deep Reefs a Potential Refuge?” Marine Ecology Progress Series 561: 217–231. 10.3354/meps11953.

[ece372044-bib-0072] McGehee, M. A. 1994. “Correspondence Between Assemblages of Coral Reef Fishes and Gradients of Water Motion, Depth, and Substrate Size Off Puerto Rico.” Marine Ecology Progress Series 105, no. 3: 243–256. 10.3354/meps105243.

[ece372044-bib-0073] Mecho, A. , B. Dewitte , J. Sellanes , S. van Gennip , E. E. Easton , and J. B. Gusmao . 2021. “Environmental Drivers of Mesophotic Echinoderm Assemblages of the Southeastern Pacific Ocean.” Frontiers in Marine Science 8, no. February: 1–15. 10.3389/fmars.2021.574780.35685121

[ece372044-bib-0074] Meekan, M. G. , A. D. L. Steven , and M. J. Fortin . 1995. “Spatial Patterns in the Distribution of Damselfishes on a Fringing Coral Reef.” Coral Reefs 14, no. 3: 151–161. 10.1007/BF00367233.

[ece372044-bib-0075] Menza, C. , M. Kendall , C. Rogers , and J. Miller . 2007. “A Deep Reef in Deep Trouble.” Continental Shelf Research 27, no. 17: 2224–2230. 10.1016/j.csr.2007.05.017.

[ece372044-bib-0076] Morato, T. , S. D. Hoyle , V. Allain , and S. J. Nicol . 2010. “Seamounts Are Hotspots of Pelagic Biodiversity in the Open Ocean.” Proceedings of the National Academy of Sciences 107, no. 21: 9707–9711. 10.1073/pnas.0910290107. PMC2906904 20448197

[ece372044-bib-0077] Núñez Lara, E. , and E. Arias González . 1998. “The Relationship Between Reef Fish Community Structure and Environmental Variables in the Southern Mexican Caribbean.” Journal of Fish Biology 53, no. Suppl. A: 209–221. 10.1006/jfbi.1998.0829.

[ece372044-bib-0078] Papastamatiou, Y. P. , C. G. Meyer , R. K. Kosaki , N. J. Wallsgrove , and B. N. Popp . 2015. “Movements and Foraging of Predators Associated With Mesophotic Coral Reefs and Their Potential for Linking Ecological Habitats.” Marine Ecology Progress Series 521: 155–170. 10.3354/meps11110.

[ece372044-bib-0079] Pereira‐Filho, G. H. , G. M. Amado‐Filho , S. M. P. B. Guimarães , et al. 2011. “Reef Fish and Benthic Assemblages of the Trindade and Martin Vaz Island Group, SouthWestern Atlantic.” Brazilian Journal of Oceanography 59, no. 3: 201–212. 10.1590/s1679-87592011000300001.

[ece372044-bib-0080] Pérez‐Rosales, G. , H. Rouzé , G. Torda , et al. 2021. “Mesophotic Coral Communities Escape Thermal Coral Bleaching in French Polynesia.” Royal Society Open Science 8, no. 11: 210139. 10.1098/rsos.210139. 34804562 PMC8580450

[ece372044-bib-0081] Pinheiro, H. T. , C. MacDonald , J. P. Quimbayo , et al. 2023. “Assembly Rules of Coral Reef Fish Communities Along the Depth Gradient.” Current Biology 33, no. 8: 1421–1430.e4. 10.1016/j.cub.2023.02.040. 36917975

[ece372044-bib-0082] Pinheiro, H. T. , C. MacDonald , R. G. Santos , et al. 2023. “Plastic Pollution on the World's Coral Reefs.” Nature 619: 311–316. 10.1038/s41586-023-06113-5. 37438592

[ece372044-bib-0083] Pitcher, T. J. , and C. Bulman . 2007. “Raiding the Larder: A Quantitative Evaluation Framework and Trophic Signature for Seamount Food Webs.” In Seamounts: Ecology, Fisheries & Conservation, edited by T. J. Pitcher , T. Morato , P. J. B. Hart , M. R. Clark , N. Haggan , and R. S. Santos , 282–295. Blackwell Publishing.

[ece372044-bib-0084] Pomar, L. , M. Morsilli , P. Hallock , and B. Bádenas . 2012. “Internal Waves, an Under‐Explored Source of Turbulence Events in the Sedimentary Record.” Earth‐Science Reviews 111, no. 1–2: 56–81. 10.1016/j.earscirev.2011.12.005.

[ece372044-bib-0085] Puglise, K. , L. M. Hinderstein , J. C. A. Marr , M. J. Dowgiallo , and F. A. Martinez . 2009. “Mesophotic Coral Ecosystems Research Strategy.” In International Workshop to Prioritize Research and Management Needs for Mesophotic Coral Ecosystems, Jupiter, Florida, 12‐15 July 2008, 24. NOAA National Centers for Coastal Ocean Science, Center for Sponsored Coastal Ocean Research, and Off. http://purl.fdlp.gov/GPO/gpo1254.

[ece372044-bib-0086] Puglise, K. A. , and P. L. Colin . 2016. “Understanding Mesophotic Coral Ecosystems: Knowledge Gaps for Management.” In Mesophotic Coral Ecosystems—A Lifeboat for Coral Reefs? edited by E. Baker , K. Puglise , and P. Harris , 83–85. United Nations Environment Programme and GRDI‐ Arendal.

[ece372044-bib-0087] Pyle, L. R. 2000. “Assessing Undiscovered Fish Biodiversity on Deep Coral Reefs Using Advanced Self‐Contained Diving Technology.” Marine Technology Society Journal 34, no. 4: 82–91.

[ece372044-bib-0088] Pyle, R. L. , and J. M. Copus . 2019. “Mesophotic Coral Ecosystems: Introduction and Overview.” In Mesophotic Coral Ecosystems. Coral Reefs of the World, edited by Y. Loya , K. Puglise , and T. Bridge , vol. 12. Springer. 10.1007/978-3-319-92735-0_1.

[ece372044-bib-0089] Pyle, R. L. , R. K. Kosaki , H. T. Pinheiro , L. A. Rocha , R. K. Whitton , and J. M. Copus . 2019. “Fishes: Biodiversity.” In Mesophotic Coral Ecosystems. Coral Reefs of the World, edited by Y. Loya , K. Puglise , and T. Bridge , vol. 12. Springer. 10.1007/978-3-319-92735-0_40.

[ece372044-bib-0090] R Core Team . 2022. R: A Language and Environment for Statistical Combbputing. R Foundation for Statistical Computing. https://www.r‐project.org/.

[ece372044-bib-0091] Riegl, B. , and W. E. Piller . 2003. “Possible Refugia for Reefs in Times of Environmental Stress.” International Journal of Earth Sciences 92, no. 4: 520–531. 10.1007/s00531-003-0328-9.

[ece372044-bib-0092] Roberts, C. , and R. Ormond . 1987. “Habitat Complexity and Coral Reef Fish Diversity and Abundance on Red Sea Fringing Reefs.” Marine Ecology Progress Series 41, no. 1: 1–8. 10.3354/meps041001.

[ece372044-bib-0093] Robinson, E. , P. Hosegood , and A. Bolton . 2024. “Modulation of the Internal Wave Regime Over a Tropical Seamount Ecosystem by Basin‐Scale Oceanographic Processes.” Progress in Oceanography 228, no. October 2023: 103323. 10.1016/j.pocean.2024.103323.

[ece372044-bib-0094] Rocha, L. A. , H. T. Pinheiro , B. Shepherd , et al. 2018. “Mesophotic Coral Ecosystems Are Threatened and Ecologically Distinct From Shallow Water Reefs.” Science 361, no. 6399: 281–284. 10.1126/science.aaq1614. 30026226

[ece372044-bib-0095] Rosa, M. R. , A. C. Alves , D. V. Medeiros , et al. 2016. “Mesophotic Reef Fish Assemblages of the Remote St. Peter and St. Paul's Archipelago, Mid‐Atlantic Ridge, Brazil.” Coral Reefs 35, no. 1: 113–123. 10.1007/s00338-015-1368-x.

[ece372044-bib-0096] Schramm, K. D. , M. J. Marnane , T. S. Elsdon , et al. 2020. “A Comparison of Stereo‐BRUVs and Stereo‐ROV Techniques for Sampling Shallow Water Fish Communities on and Off Pipelines.” Marine Environmental Research 162, no. October: 105198. 10.1016/j.marenvres.2020.105198. 33130445

[ece372044-bib-0097] Scott, M. E. , S. B. Tebbett , K. L. Whitman , et al. 2022. “Variation in Abundance, Diversity and Composition of Coral Reef Fishes With Increasing Depth at a Submerged Shoal in the Northern Great Barrier Reef.” Reviews in Fish Biology and Fisheries 32, no. 3: 941–962. 10.1007/s11160-022-09716-9.

[ece372044-bib-0098] Sih, T. L. , M. Cappo , and M. Kingsford . 2017. “Deep‐Reef Fish Assemblages of the Great Barrier Reef Shelf‐Break (Australia).” Scientific Reports 7, no. 1: 10886. 10.1038/s41598-017-11452-1. 28883506 PMC5589835

[ece372044-bib-0099] Srinivasan, M. 2003. “Depth Distributions of Coral Reef Fishes: The Influence of Microhabitat Structure, Settlement, and Post‐Settlement Processes.” Oecologia 137, no. 1: 76–84. 10.1007/s00442-003-1320-6. 12856204

[ece372044-bib-0100] Stan Development Team . 2024. “RStan: The R Interface to Stan.” R Package Version 2.32.6. https://mc‐stan.org/.

[ece372044-bib-0101] Sward, D. , J. Monk , and N. Barrett . 2019. “A Systematic Review of Remotely Operated Vehicle Surveys for Visually Assessing Fish Assemblages.” Frontiers in Marine Science 6, no. APR: 1–19. 10.3389/fmars.2019.00134.36817748

[ece372044-bib-0102] Thompson, C. D. H. , J. J. Meeuwig , A. M. Friedlander , and E. Sala . 2024. “Remote Seamounts Are Key Conservation Priorities for Pelagic Wildlife.” Conservation Letters 17: 1–4. 10.1111/conl.12993.

[ece372044-bib-0103] Thresher, R. E. , and P. L. Colin . 1986. “Trophic Structure, Diversity and Abundance of Fishes of the Deep Reef (30‐300 m) at Enewetak, Marshall Islands.” Bulletin of Marine Science 38, no. 1: 253–272.

[ece372044-bib-0104] Van den Hoek, C. , A. M. Breeman , R. P. M. Bak , and G. Van Buurt . 1978. “The Distribution of Algae, Corals and Gorgonians in Relation to Depth, Light Attenuation, Water Movement and Grazing Pressure in the Fringing Coral Reef of Curaçao, Netherlands Antilles.” Aquatic Botany 5, no. C: 1–46. 10.1016/0304-3770(78)90045-1.

[ece372044-bib-0105] Vehtari, A. 2023. “loo: Efficient Leave‐One‐Out Cross‐Validation and WAIC for Bayesian Models.” R Package Version 2.6.0. https://mc‐stan.org/loo/.

[ece372044-bib-0106] Waldner, R. E. , and D. R. Robertson . 1980. “Patterns of Habitat Partitioning by Eight Species of Territorial Caribbean Damselfishes (Pisces: Pomacentridae).” Bulletin of Marine Science 30, no. 1978: 171–186.

[ece372044-bib-0107] Wang, Y. , U. Naumann , D. Eddelbuettel , et al. 2022. “mvabund: Statistical Methods for Analysing Multivariate Abundance Data.” https://rdrr.io/cran/mvabund/.

[ece372044-bib-0108] Wen, C. K. C. , M. S. Pratchett , G. R. Almany , and G. P. Jones . 2013. “Patterns of Recruitment and Microhabitat Associations for Three Predatory Coral Reef Fishes on the Southern Great Barrier Reef, Australia.” Coral Reefs 32, no. 2: 389–398. 10.1007/s00338-012-0985-x.

[ece372044-bib-0109] White, M. , I. Bashmachnikov , J. Arístegui , and A. Martins . 2007. “Physical Processes and Seamount Productivity.” In Seamounts: Ecology, Fisheries & Conservation, edited by T. J. Pitcher , T. Morato , P. J. B. Hart , M. R. Clark , N. Haggan , and R. S. Santos , 62–84. Blackwell Publishing.

[ece372044-bib-0110] Wickham, H. , M. Averick , J. Bryan , et al. 2019. “Welcome to the Tidyverse.” Journal of Open Source Software 4: 1686. 10.21105/JOSS.01686.

[ece372044-bib-0111] Williams, D. M. 1991. “Patterns and Processes in the Distribution of Coral Reef Fishes.” In The Ecology of Fishes on Coral Reefs, edited by P. F. Sale , 437–474. Academic Press.

[ece372044-bib-0112] Willig, M. R. , and S. J. Presley . 2016. “Biodiversity and Metacommunity Structure of Animals Along Altitudinal Gradients in Tropical Montane Forests.” Journal of Tropical Ecology 32, no. 5: 421–436. 10.1017/S0266467415000589.

[ece372044-bib-0113] Wright, D. H. 1983. “Species‐Energy Theory: An Extension of Species‐Area Theory.” Oikos 41, no. 3: 496–506.

